# Segmental resection with primary anastomosis is not always safe in splenic flexure perforation

**DOI:** 10.1186/s13104-016-1841-9

**Published:** 2016-01-16

**Authors:** Elroy P. Weledji, Martin D. Mokake, Motaze Sinju

**Affiliations:** Faculty of Health Sciences, University of Buea, P.O. Box 126, Limbe, S.W. Region, Buea, Cameroon; Regional Hospital Buea, S.W. Region, Buea, Cameroon

**Keywords:** Familial adenomatous polyposis, Perforation, Splenic flexure, Resection, Anastomosis, Stoma

## Abstract

**Background:**

Familial adenomatous polyposis (FAP) is caused by a rare mutation of the adenomatous polyposis coli gene on Chromosome 5q. The risk of colorectal cancer in patients with FAP is nearly 100 % and intensive endoscopic surveillance or prophylactic colectomy are mandatory. If extensive endoscopic surveillance is chosen, there is a cumulative risk of perforation and bleeding especially after polypectomy. We discussed the problems and options in the management of the late diagnosis of an iatrogenic perforation of the splenic flexure complicating endoscopic surveillance in FAP.

**Case presentation:**

We present a 35-year-old black African man with FAP who sustained a splenic flexure perforation following a colonoscopic polypectomy of a suspicious lesion. He underwent a splenic flexure resection and primary anastomosis that dehisced and the patient benefited from an emergency definitive colectomy and ileorectal anastomosis.

**Conclusions:**

Resection with primary anastomosis following iatrogenic perforation of the splenic flexure is not safe because of a high chance of anastomotic dehiscence. Following a late diagnosis in an unstable patient exteriorization of the perforation as a stoma is a better option prior to a definitive prophylactic colectomy.

## Background

Familial adenomatous polyposis (FAP) is caused by a rare mutation with the frequency in the general population in the west being 1:13,528 [1]. It is caused by the germ-line mutation of the tumour suppressor APC gene on chromosome 5q. It is of autosomal dominance inheritance with offspring of affected individuals having a 1 in 2 chance of inheriting FAP [2, 3]. Genotype–phenotype correlations have distinguished the severe FAP with dense colorectal polyposis at a young age (second or third decade of life) and relatively early colorectal cancer development, duodenal adenomatous polyps and multiple extraintestinal manifestations from less severe FAP (‘attenuated polyposis’) which would guide surveillance and treatment [4]. The attenuated adenomatous polyposis may be overlooked in asymptomatic family members with few adenomas and low expressivity of the gene defect. Other modifier genes and the environment may play a role in disease expression. The risk of colorectal cancer in patients with FAP is nearly 100 and 7 % risk of gastroduodenal cancer [5]. The results of endoscopic surveillance of FAP patients demonstrated a cumulative risk of 2.8 % for perforation, 11 % for serious bleeding, and 0.05 % for procedure-related death. High rates are associated with polypectomy [6]. Although life expectancy in patients with FAP is still less than that of the general population, prophylactic surgery (colectomy and ileorectal anastomosis or a restorative proctocolectomy) has improved survival [7, 8]. We discussed the problems and options in the management of the late diagnosis of an iatrogenic perforation complicating endoscopic surveillance in FAP.

## Case presentation

**Fig. 1:**
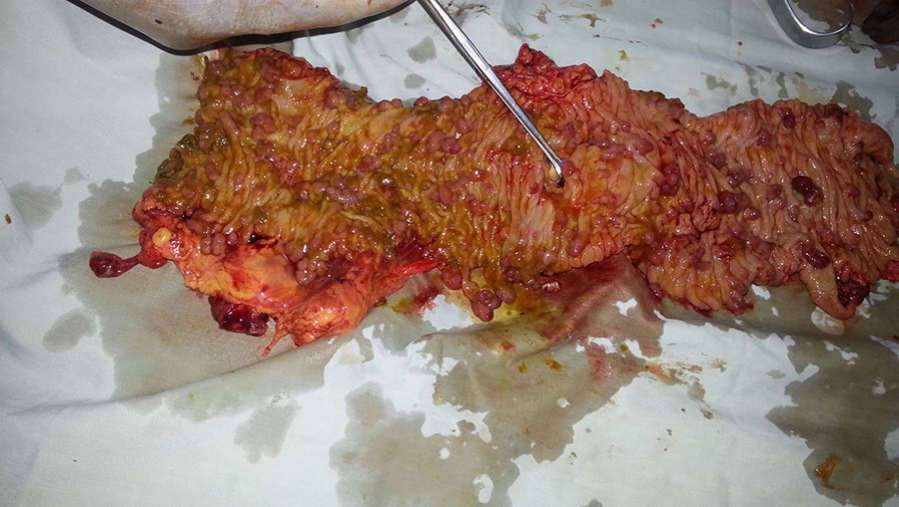
Segmental resection specimen demonstrating splenic flexure carpeted with polyps and site of perforation

**Fig. 2:**
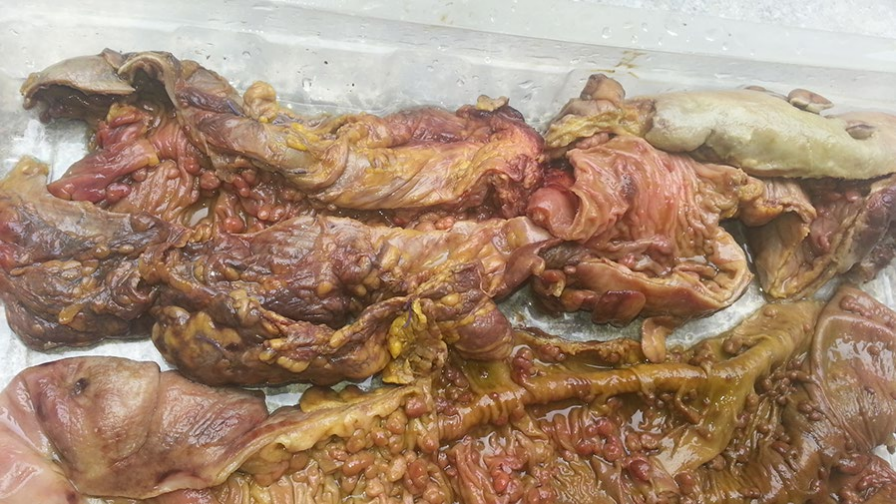
Total colectomy specimen demonstrating caecum and ascending colon (*below*) and descending colon above carpeted with polyps with site of previous anastomosis at centre (*above*)

**Fig. 3:**
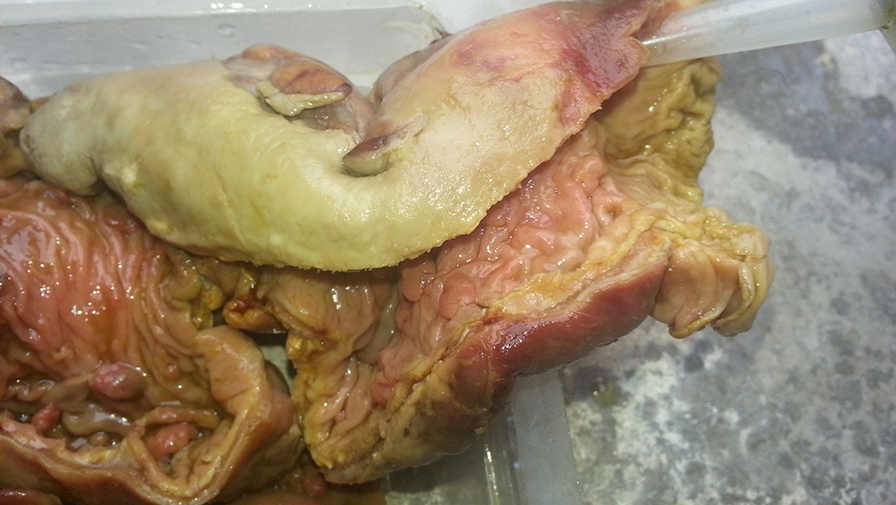
Colectomy specimen demonstrating no polyp in the distal sigmoid colon and rectum

A 35-year-old black African man with known FAP was admitted as an emergency with an acute abdomen two weeks after a colonoscopic polypectomy of an apparently suspicious lesion. The abdominal pain became evident 2 days following the procedure. It was constant and progressive and gradually associated with abdominal distension. There was no vomiting but decreased stool frequency. An abdominal ultrasound scan had revealed small bowel obstruction. He was diagnosed FAP at age 25 years following a history of recurring abdominal pain, rectal bleeding and diarrhea alternating with constipation. His mother died from colon cancer aged 60. He has a 30-year-old sister who has not been screened but otherwise well. He gave a history of appendicectomy 8 years back. On examination the patient was in great distress, lying still with a centrally distended abdomen and chest movements only. The vital signs revealed a BP 96/65 mmHg, HR 120/min, RR 24/min and temperature of 39.1 °C. There was abdominal guarding, a silent abdomen and generalized rebound tenderness. A clinical diagnosis of peritonitis from an iatrogenic colonic perforation was made. He was kept nil by mouth, and resuscitated with intravenous fluids and antibiotics. His haemoglobin level was 7.1 g/l, WBC 13 × 10^9^/l, (N:4–10) Pts 301 × 10^9^/l (150–400). A low MCV 54.9 fl (80–100) was consistent with a microcytic hypochromic anaemia for which he received two units of blood. He refused consent for a subtotal colectomy with a temporary ileostomy but accepted a laparotomy with the possibility of primary closure of the perforation so as to resolve the immediate problem of sepsis. At laparotomy, there was localized purulent peritonitis and distended loops of small bowel adherent to an inflamed splenic flexure colonic mass. A difficult splenic flexure resection was done extending from mid transverse colon to upper descending colon (Fig. 1). An end-to-end anastomosis with 2-0 vicryl used an interrupted inverting vertical mattress suturing of the posterior layer and a sero-submucosal suturing of the anterior layer. This rendered good mucosal apposition and avoided the interfering polyps at the edges. On the 5th postoperative day he developed signs of generalized peritonitis with a leukocytosis of 19 × 10^9^ cells/l and a pyrexia of 38.5 °C but haemodynamically stable. A second emergency laparotomy revealed a faecal peritonitis from anastomotic dehiscence. He underwent a subtotal colectomy with an ileorectal anastomosis (Figs. 2 and 3). The procedure lasted 3 h and the patient lost less than 200 ml of blood and received a unit of blood perioperatively. He made rapid recovery with bowel movement the following day. On the 6th postoperative day he developed a low output enterocutaneous fistula from a complication of the pelvic drain which was managed conservatively. His stool frequency was thrice per day on discharge and a follow-up six monthly surveillance was planned.

## Discussion

**Fig. 4:**
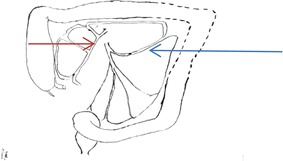
Schematic diagram of the vascular supply of the splenic flexure (Griffith’s point supplies the *dotted line* area (splenic flexure). *Blue arrow—*left colic artery from inferior mesenteric artery (divide there to support the marginal artery at the splenic flexure); *Brown arrow—*superior mesenteric artery giving off middle colic artery

The first report of FAP was by Skifosovski in 1881 [9]. Although FAP accounts for less than 1 % of all colorectal cancers it has provided knowledge about carcinogenesis and colon cancer. The average diagnosed patient with FAP already had cancer by aged 39 years, approximately 25 years earlier than in the general population. The implication is that treatment should be by age 20 years [4, 10]. Colonoscopy is used for diagnosis, screening and biopsy [6]. There is a small risk of colonic perforation which may be noted during the procedure or it may only be diagnosed hours or even days after the procedure as in this case. If the perforation is noted at colonoscopy or if there are signs of a generalized or spreading peritonitis, immediate laparotomy is indicated with primary closure of the perforation. Perforation not associated with signs of peritonitis may be managed conservatively with antibiotics and intravenous fluids, even in the presence of free gas on an abdominal radiograph [11]. When diagnosed late the wound is larger, chronically inflamed with associated localized sepsis and frank peritonitis. As in this case it could not be closed primarily and the better options were for a subtotal colectomy and ileostomy or exteriorization as a stoma. As the patient refused consent for these a segmental resection was performed [12, 13]. The largest experience in the management of penetrating colon injuries by resection with primary anastomosis demonstrated a 14 % incidence of anastomotic leak. Chronic disease or massive blood loss (or both) was associated with anastomotic leak in 42 % of patients [14]. This case demonstrated the dilemma in the management of a late diagnosis of iatrogenic perforation in FAP in a difficult patient. It was accentuated by the splenic flexure site of the perforation which normally has a marginal blood supply between the terminal two branches of the left colic artery (Griffith’s point) (Fig. 4). It is important when mobilizing the blood supply at the splenic flexure to preserve these two branches so as to support the marginal artery at this point but, in this case the vascular anatomy would have been distorted from the densely inflamed splenic flexure mass [15].

A series of cases of duodenal perforation following polypectomy in FAP were managed successfully with omental patch closure following early diagnosis but there has been no report in the literature on the management of a late diagnosis of an iatrogenic perforation [16]. For source control of sepsis, the well-known principle in the management of a perforated chronically diseased colon as in severe ulcerative colitis, toxic megacolon from infective colitis even in the in the unstable patient, is a rapid subtotal colectomy with preservation of the rectal stump and formation of a terminal ileostomy. This would later allow the option of an ileorectal anastomosis or construction of an ileo-anal pouch after a completion protectomy once the sepsis has resolved [10, 17]. This may also hold for sepsis complicating an iatrogenic colonic perforation in FAP. However, light clothing, hot climate, high residue diet (vegetables), poor availability of appliances, sepsis-induced high ileostomy output and cultural taboos make the management of an ileostomy more difficult in the tropics, and so ileorectal anastomosis with endoscopic surveillance is preferred unless the rectum is extensively diseased [18, 19]. Another option would have been to exteriorize the perforation as a double-barrelled stoma (Paul Mikulicz procedure) [14, 20]. It is also of note that an extended hemicolectomy or subtotal colectomy are oncologically and anastomotically safe procedures for a spontaneously perforating splenic flexure carcinoma [10].

In the second laparotomy the patient was more stable as a result of the early diagnosis of the postoperative peritonitis, so he could undergo the longer and more extensive surgery of total colectomy and ileorectal anastomosis. In this case the rectum was free of polyps (Fig. 3) and the one layer, interrupted inverting serosubmucosal suturing technique was used for its adaptability to any anastomosis involving the colon with leak rates of 0.5–3 % in sizeable series [21, 22]. The advantages of IRA are that it is a one-stage procedure with lower morbidity and mortality, whereas, restorative proctocolectomy (RPC) often involves a temporary defunctioning ileostomy. The functional results in terms of stool frequency and leakage are generally slightly better than after RPC [10]. Although pouchitis is rare in FAP, the biggest attraction of RPC is that there is no risk of polyps developing in a retained rectum. However follow-up studies have shown adenomas developing in the ileal pouch and along with a pouch failure rate of 10 % from pelvic sepsis and poor function these may result in the need for a permanent ileostomy [23, 24]. Follow-up peranal digital and flexible endoscopic examination are mandatory after IRA or RPC [24, 25].

## Conclusions

Following a colonoscopic perforation the management depends on the damage to the bowel, the site in the bowel and the patient’s health status. The options of a splenic flexure perforation are local repair, segmental resection or subtotal colectomy with or without a stoma. In this case the option of segmental resection was followed by anastomotic dehiscence and the subsequent subtotal colectomy and ileo-rectal anastomosis was successful. Segmental resection and anastomosis is not always safe for splenic flexure perforation. Following a late diagnosis in an unstable patient exteriorization of the perforation as a stoma is a simpler and better option prior to a definitive elective colectomy for FAP. For early diagnosis with peritonitis in a stable patient a subtotal colectomy would allow the option of an ileorectal anastomosis or the construction of an ileo-anal pouch once the sepsis has resolved.

## Consent

Written informed consent was obtained from the patient for publication of this Case report and any accompanying images.

## Abbreviations

FAP: familial adenomatous polyposis
APC: adenomatous polyposis gene
IRA: ileorectal anastomosis
RPC: restorative proctocolectomy

## References

[1] Bulow S (1989). Familial adenomatous polyposis. Ann Med.

[2] Gardner EJ (1951). A genetic and clinical study of intestinal polyposis, a predisposing factor for carcinoma of the colon and rectum. Am J Hum Genet.

[3] Bisgaard ML, Fenger K, Bulow S, Niebur E, Mohr J (1994). Familial adenomatous polyposis (FAP): frequency, penetrance and mutation rate. Human Mutat.

[4] Wu JS, Paul P, McGannon EA, Church JM (1998). APC genotype, polyp number, and surgical options in familial adenomatous polyposis. Ann Surg.

[5] Crabtree MD, Tomlinson IPM, Hodgson SV, Neale K, Phillips KK, Houlston RS (2002). Explaining variation in familial adenomatous polyposis: relationship between genotype and phenotype and evidence for modifier genes. Gut.

[6] Waye JD, Lewis BS, Yessayan S (1992). Colonoscopy: a prospective report of complications. Lancet.

[7] Belchetz LA, Berk T, Bapat BV, Cohen Z, Gallinger S (1996). Changing causes of mortality in patients with familial adenomatous polyposis. Dis Colon Rectum.

[8] Tudyka VN, Clark SK (2012). Surgical treatment in familial adenomatous polyposis. Ann Gastroenterol..

[9] Sklifosovski NV (1881). Polyadenoma tractus intestinalis. Vrach.

[10] Nugent KP, Northover J, Phillips RKS, Spibelman AD, Thomson JPS (1994). Total colectomy and ileorectal anastomosis. Familial adenomatous polyposis ansd other polyposis syndromes.

[11] Donkier V, Andre R (1993). Treatment of colon endoscopic perforations. Acta Chir Belg.

[12] Sasaki LS, Allaben RD, Golwak R, Mital VK (1995). Primary repair of colon injuries: a prospective randomized study. J Trauma.

[13] Chappius CW, Frey DJ, Dietzen CD, Panetta TP, Buechter KJ, Colin I (1991). Management of penetrating colon injuries: a prospective randomized trial. Ann Surg.

[14] Stewart RM, Fabian TC, Croce MA, Pritchard FE, Minard G, Kudsk KA (1994). Is resection with primary anastomosis following destructive colon wounds always safe?. Am J Surg.

[15] Griffiths JD (1956). Surgical anatomy of the blood supply of the distal colon. Ann R Coll Surg Engl.

[16] Penna C, Phillips RK, Tiret E, Spigelman AD (1993). Surgical polypectomy of duodenal adenomas in familial adenomatous polyposis: experience of two European centres. Br J Surg.

[17] Melville DM, Ritchie JK, Nicholls RJ, Hawley PR (1994). Surgery for ulcerative colitis in the era of the pouch: the St Mark’s Hospital experience. Gut.

[18] Campos FG (2014). Surgical treatment of familial adenomatous polyposis: dilemmas and current recommendations. World J Gastroenterol.

[19] Roy D, Taylor S, Chisholm GD, O’Higgins N, Shields R (1990). Surgery in tropical countries. Surgical management.

[20] Nance FC (1995). A stake through the heart of colostomy. J Trauma.

[21] Carry NJ, Keating J, Campbell J (1991). Prospective audit of an extramucosal technique for intestinal anastomosis. Br J Surg.

[22] Matheson NA, McIntosh CA, Krukowski ZH (1985). Continuing experience with single layer appositional anastomosis in the large bowel. Br J Surg.

[23] Aziz O, Athanasiou T, Fazio VW, Nicholls RJ, Darzi AW, Church J, Phillips RK, Tekkis PP (2006). Meta analysis of observational studies of ileorectal versus ileal pouch anal anastomosis for familial adenomatous polyposis. Br J Surg.

[24] M’Koma AE, Herline AJ, Adunyah SE. Subsequent adenomas of ileal pouch and anorectal segment after prophylactic surgery for familial adenomatous polyposis. World J Colorectal Surg. 2013;3(2):art1. PMC4012278 24817992

[25] Church J, Burke C, McGammon E, Pastean O, Clark B (2003). Risk of rectal cancer after colectomy and ileorectal anastomosis for familial adenomatous polyposis: a function of available options. Dis Colon Rectum.

